# Feasibility and applicability of the paper and electronic COPD assessment test (CAT) and the clinical COPD questionnaire (CCQ) in primary care: a clinimetric study

**DOI:** 10.1038/s41533-017-0023-0

**Published:** 2017-03-28

**Authors:** J. W. H. Kocks, C. M. G. Blom, M. J. Kasteleyn, W. Oosterom, B. J. Kollen, T. Van der Molen, N. H. Chavannes

**Affiliations:** 1 Department of General Practice, University of Groningen, University Medical Center Groningen, Groningen, The Netherlands; 2 GRIAC Research Institute, University of Groningen, University Medical Center Groningen, Groningen, The Netherlands; 3 Zorgdraad Foundation, Oosterbeek, The Netherlands; 4 Department of Public Health and Primary Care LUMC Leiden, Leiden, The Netherlands; 5 Department of Pulmonology, LUMC Leiden, Leiden, The Netherlands

## Abstract

Three questionnaires are recommended in the management of chronic obstructive pulmonary disease by the global initiative for obstructive lung disease, of which two are the more comprehensive assessments: the chronic obstructive pulmonary disease assessment test and the clinical chronic obstructive pulmonary disease questionnaire. Both are carefully designed high-quality questionnaires, but information on the feasibility for routine use is scarce. The aim of this study was to compare the time to complete the chronic obstructive pulmonary disease assessment test and the clinical chronic obstructive pulmonary disease questionnaire and the acceptability of the questionnaires. Furthermore, the agreement between electronic and paper versions of the questionnaires was explored. The time to complete the electronic versions of the questionnaires was 99.6 [IQR 74; 157] vs. 97.5 [IQR 68; 136] seconds for clinical clinical chronic obstructive pulmonary disease questionnaire and chronic obstructive pulmonary disease assessment test, respectively. The difference in time to complete the questionnaire was not significant. The two questionnaires did not differ in “easiness to complete” or “importance of issues raised in questionnaires”. Electronic vs. paper versions revealed high agreement (ICC CCQ = 0.815 [0.712; 0.883] and ICC CAT = 0.751 [0.608; 0.847]) between the administration methods. Based on this study it can be concluded that both questionnaires are equally suitable for use in routine clinical practice, because they are both quick to complete and have a good acceptability by the patient. Agreement between electronic and paper versions of the questionnaires was high, so use of electronic versions is justified.

## COPD: questionnaires equally suitable for clinical practice

Two questionnaires commonly used to manage chronic obstructive pulmonary diseases (COPD) are equally suitable for routine primary care. Researchers in The Netherlands, led by Janwillem Kocks from the University Medical Center Groningen, administered both the COPD assessment test (CAT) and the clinical COPD questionnaire (CCQ) to 95 patients with the lung disease. These two tests are the most comprehensive assessments recommended by the global initiative for obstructive lung disease for guiding treatment decisions. The researchers found that both tests took approximately 95–100 s on average. Both tests were also equally easy to complete and provided similar types of information. Most patients said they had no preference for either one, and they filled out both electronic and paper versions of the questionnaires in much the same way. The authors conclude that both tests seem fine for routine use.

## Introduction

Questionnaires are recommended in the management of chronic obstructive pulmonary disease (COPD).^1, 2^ Over the last years several compact questionnaires addressing health status have been specifically designed to be used in routine clinical practice. The use of these questionnaires is thought to improve communication^3^ and can guide treatment.^2, 4^

Since 2011, the global initiative for obstructive lung disease (GOLD) guidelines/strategy^5^ has included three questionnaires in the assessment of COPD patients: the modified Medical Research Council (mMRC) dyspnea scale,^6^ the COPD assessment test (CAT),^3^ and the clinical COPD questionnaire (CCQ).^7^ As the mMRC solely addresses dyspnea, the CAT and CCQ are the more comprehensive assessments of the three questionnaires providing the clinician with more valuable information regarding burden of disease. For that reason we focus in this study on the head-to-head comparison between CAT and CCQ. The CAT consists of eight items scored on a 5-point scale and a total score can be calculated. The CCQ consists of 10 items scored on a 6-point scale, and a total score as well as symptom, functional, and mental status domain scores can be calculated.

Guideline developers and clinicians need to make choices on which of these two health status questionnaires they should recommend and use in daily practice. Next to the choices that have to be made on the content, quality, responsiveness, and comparability of these questionnaires, the feasibility for actual use in clinical practice is essential for successful implementation.

To date, only scarce information is available that compares the feasibility of the CAT and the CCQ. We studied both questionnaires regarding the average time to complete and acceptability. Furthermore, agreement between electronic and paper versions of both questionnaires was explored.

## Results

**Fig. 1:**
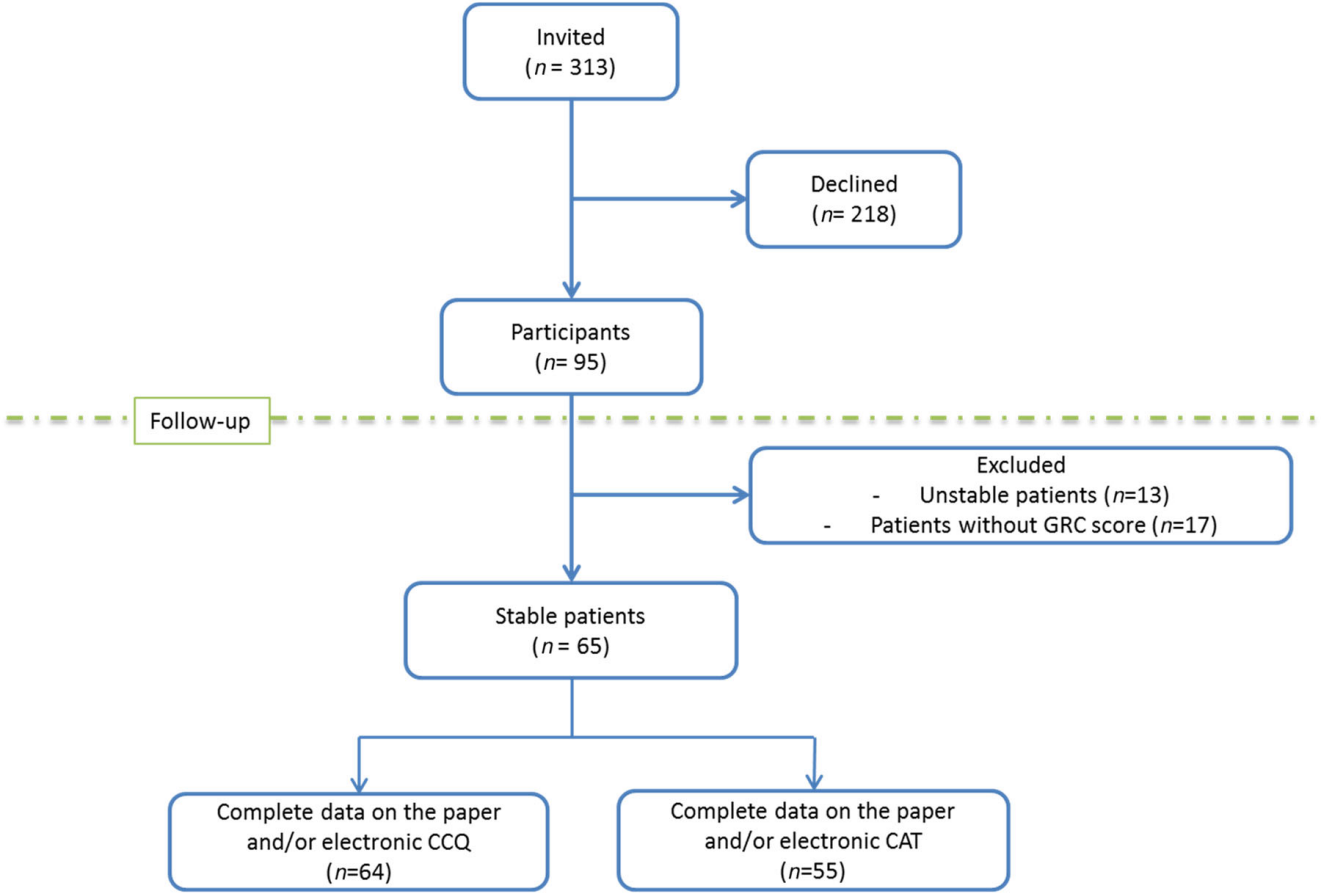
Flowchart of the study. *GRC* global rating of change, *CCQ* clinical COPD questionnaire, *CAT* COPD assessment test

COPD patients were invited to the study (Fig. 1). In total, 95 COPD patients participated in the study and completed the questionnaires online. Baseline characteristics are presented in Table 1. The total scores could not be calculated in 10% of CAT, and in 1% of the CCQ and mMRC due to missing values.

**Table 1:** Baseline characteristics

	COPD patients (*n* = 95)
Age in years, mean (SD)	65.0 (10.0)
Male gender, *n* (%)	60 (63.2)
CAT score, mean (SD)	13.2 (7.4)
CCQ score, mean (SD)	1.6 (1.0)
mMRC score, median [IQR]	1.0 [1.0–2.0]

*Note*: Normal distributed variables are presented as mean (SD), non-normal variables as median [IQR]

*COPD* chronic obstructive pulmonary disease, *CCQ* clinical COPD questionnaire, *CAT* COPD assessment test, *mMRC* modified Medical Research Council

### Differences between electronic version of the CAT and CCQ

The differences between the electronic version of the CAT and CCQ were tested in 95 patients and reported in Table 2. The median CAT completion time was 97.5 [IQR 68–136] vs. a median CCQ completion time of 99.6 [IQR 74–157] seconds (*p* = 0.151).

**Table 2:** Differences between electronic version of the CAT and the electronic version of the CCQ

	CCQ	CAT	*p*-value
Completion time in seconds, median [IQR]^a^	99.6 [74–157]	97.5 [68–136]	0.151
Easiness to complete (0–10), median [IQR]^b^	8.0 [5–10]	7.5 [5–9]	0.109
Importance of issues raised (0–10), median [IQR]^b^	5.0 [5–7]	5.0 [5–7]	0.543
Importance of information for health care provider (0–10), median [IQR]^a^	7.0 [5–8]	8.0 [5–8]	0.836

*COPD* chronic obstructive pulmonary disease, *CCQ* clinical COPD questionnaire, *CAT* COPD assessment test, *mMRC* modified Medical Research Council

^a^ Analyzed using paired sample *t*-test based on logtransformed variables

^b^ Analyzed using Wilcoxon signed-rank tests

The easiness to complete, importance of issues raised in questionnaires, and the importance of information for health-care provider were not different between the two questionnaires.

Of the participants who completed the question on questionnaire preference (*n* = 78), the majority (79.5%) had no preference for one of the questionnaires, while 16.7% preferred the CCQ and 3.8% preferred the CAT.

### Agreement between electronic and paper versions of the CAT and CCQ

**Fig. 2:**
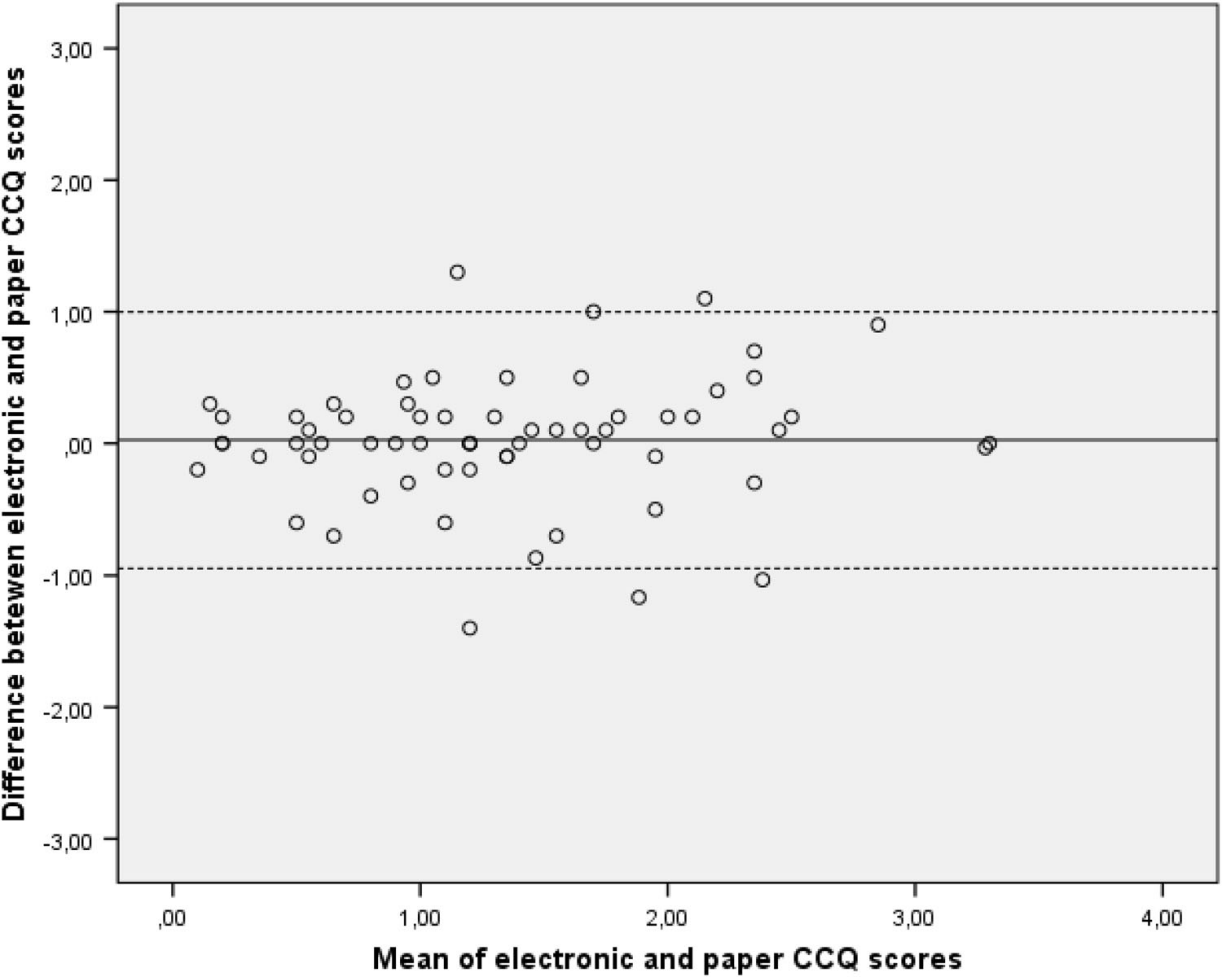
Bland–Altman plot showing the relationship between the electronic and paper version of the CCQ. The *dashed lines* represent the limits of agreement

**Fig. 3:**
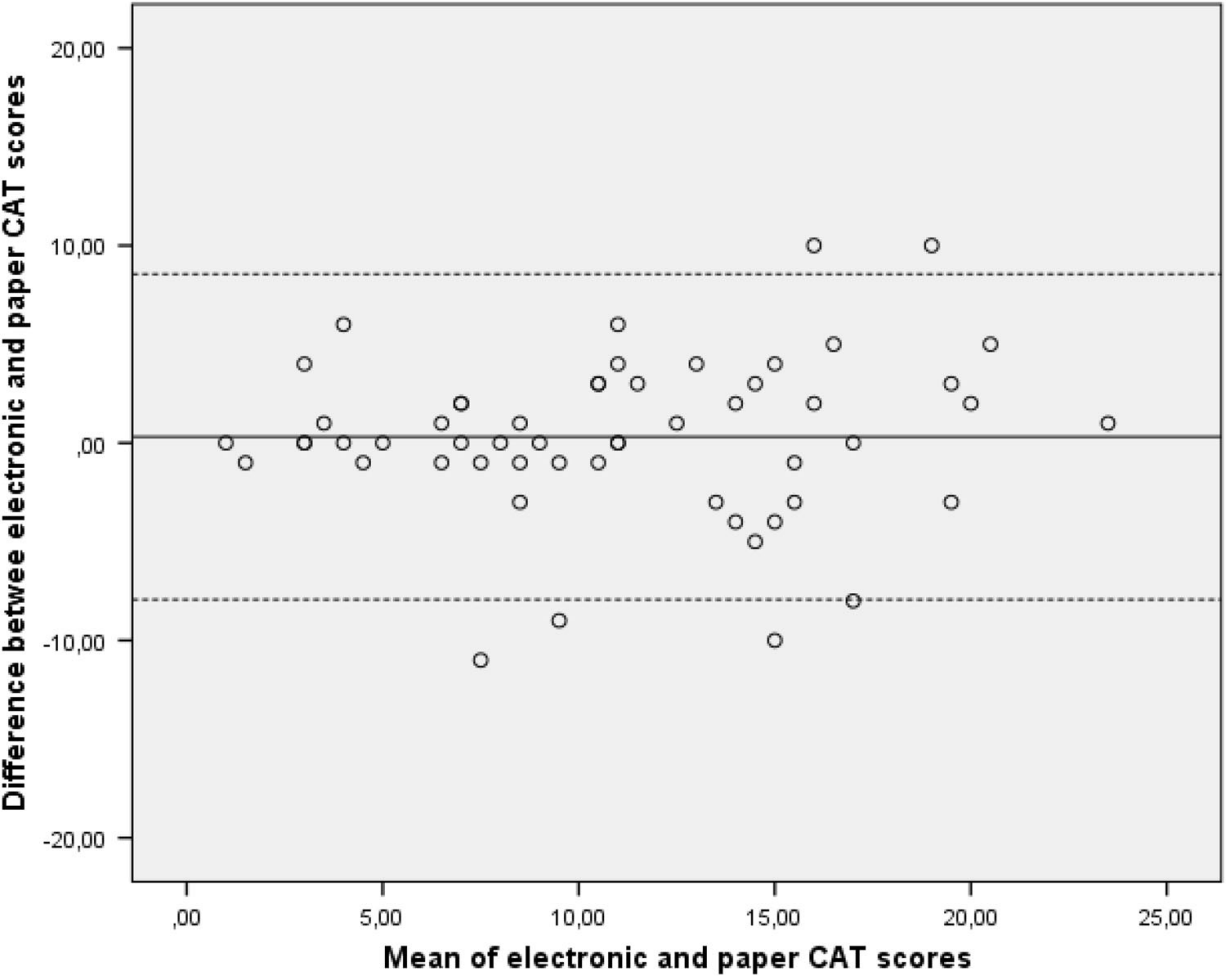
Bland–Altman plot showing the relationship between the electronic and paper version of the CAT. The *dashed lines* represent the limits of agreement

For comparison of the electronic and paper versions, only stable patients (*n* = 65) were included in the analysis. Of those, 64 had no missing data on the electronic or paper version of the CCQ and 55 had no missing data on the CAT. The absolute agreement between electronic and paper versions was high (ICC [95% CI] CCQ=0.815 [0.712; 0.883] and ICC [95% CI] CAT=0.751 [0.608; 0.847]). Interpretation of Bland–Altman plots essentially supports agreement between the two versions (Figs 2, 3).

## Discussion

### Main findings

To the best of our knowledge, this is the first study comparing CCQ and CAT head-to-head in a primary care population to assess completion time and ease of use. This study demonstrates that in a primary care COPD population neither statistically nor clinically relevant differences in filling out time or acceptability between the two health status questionnaires were observed. Agreement between electronic and paper versions of the questionnaires was high.

### Interpretation of findings in relation to previously published work

One earlier study by Ringbaek^8^ in a group of mainly severe COPD patients participating in a pulmonary rehabilitation program found patients needed 107 s to complete CAT, and 134 s to complete CCQ, but did not report whether this difference was significant. They also found that the need for assistance while answering the questionnaire was 53.9% for CAT, and 36.0% for CCQ. In our study, no assistance was given, but in the CAT questionnaire more questions remained unanswered than in the CCQ (10 vs. 1%). In contrast, Sundh et al. found a considerably higher proportion of the patients were able to complete the CAT compared with the CCQ,^9^ although they found that a slight majority of the patients preferred the CCQ.

In a study by Tsiligianni and colleagues,^10^ 10% of the patients reported that the CCQ was easier to understand than the CAT. However, 62% indicated that the CCQ reflected their health status better than the CAT did because the CCQ addresses their breathing problems better while sleep was less important to them.^10^ In our study no differences were found regarding easiness to complete the questionnaires or the importance of the topics addressed between the questionnaires.

The agreement between electronic and paper versions of both CAT and CCQ was high, which justifies electronic use in daily practice or by patients themselves online.

The decision on which health status questionnaire to use can be made based on local preferences, or based on the fact that in addition to the total score the CCQ offers three subdomain scores (symptom, functional, and mental status) that can be used to guide treatment prioritization in practice. Furthermore, the usefulness of the questionnaires from a clinician’s perspective, including the ease of deriving a score, should be taken into account. Also the impact of missing items on the score or the impact of remotely completing the questionnaires should be considered. However, that was not within the scope of this study. This study adds to our knowledge the acceptance and feasibility for the patients in addition to the known feasibility for primary care as assessed by the IPCRG.^11^

### Strengths and limitations of this study

A limitation of this study is that we could not rule out selection bias. Patients are invited to participate, and non-response or reasons for not participating are not examined. Moreover, characteristics between participants and non-participants could not be compared. Selection bias might reduce generalizability. Although it can be thought that factors like COPD severity, age, or comorbidity might influence time to complete the questionnaire, we expect that this will be the same for both questionnaires.

Importance of topics addressed is more likely to depend on severity, since more severe patients might have other or more symptoms than less severe patients. Our population is relatively mild based on mMRC, CAT, and CCQ scores. We cannot be sure whether the results of this study on importance of the topics addressed in the questionnaires can be generalized to more severe populations.

Another factor that might influence the results, especially easiness of the questions, is socio-economic status (SES). Participants are included from different parts of the Netherlands and from different general practices. Nevertheless, we cannot be sure that we included patients from with different SES levels.

Finally, the questions regarding acceptability of the CAT and CCQ were not pilot tested. Nevertheless, those questions were quite straight forward and the majority of the patients completed those questions. Because the same questions were used for both questionnaires we believe it is legitimate to draw conclusions based on those questions.

### Implications for future research, policy, and practice

Based on this study, both questionnaires seem equally suitable for use in routine clinical practice, because they are both quick to complete and have a good acceptability by the patient.

### Conclusions

No significant differences were found in the completion times and acceptability between the CCQ and the CAT in Dutch primary care. Based on this study it can be concluded that both questionnaires are equally suitable for use in routine clinical practice, because they are both quick to complete and have a good acceptability by the patient. Agreement between electronic and paper versions of the questionnaires was high, so use of electronic versions is justified.

## Methods

### Patients

COPD patients with a doctors’ diagnosis of COPD according to current guidelines^12^ were recruited from general practices and primary care rehabilitation physiotherapy programs in and around the Groningen and Rotterdam areas in the Netherlands. Inclusion criteria were (1) a doctor’s diagnosed COPD and (2) electronic informed consent. Exclusion criteria were (1) inability to understand or read the Dutch language and (2) unable to connect to the internet.

Primary care practitioners identified patients with COPD and invited them by letter or in person to participate in the study.

Participants logged in on the website to sign an electronic informed consent form and start with the study.

### Outcomes

The primary outcome was the time (in seconds) required to complete the electronic versions of the CAT and CCQ questionnaire. The time running from showing the questionnaire on the screen until clicking “completed, next page” was recorded.

The CCQ is an instrument to measure health status in patients with COPD.^7^ It consists of 10 questions on three domains: symptoms, mental state, and functional state. The symptoms and functional state domains contain four items each, and the mental state domain two. Questions are scored on a 7-point scale from 0–6, with 0 representing the best possible score and 6 representing the worst possible score. Total scores range from 0–6. A higher CCQ value indicates a lower health status.

The CAT also measures health status in patients with COPD. The CAT has eight items and includes questions about symptoms, energy, sleep, and activity.^3^ Total scores range from 0 to 40, where 0 represents no impairment.

Secondary outcomes were the degree of acceptability, measured with three questions on easiness (How difficult was completing the questionnaire?), importance of issues raised in questionnaires (To what extent did you feel the questionnaire addressed all aspects of your disease?) and importance of information for health-care provider (To what degree do you think that the questionnaire gives relevant information about your disease to your doctor?), scored on a visual analog scales ranging from 0 to 10, where 0 indicates a low acceptability and 10 indicates high acceptability. In addition, patients were asked to indicate which questionnaire they would recommend to their general practitioner.

The MRC questionnaire is a one-dimensional tool to measure dyspnea during exercise in five levels (range 0–5).^6^ The global rating of change (GRC) questionnaire was used to assess change in breathlessness between completing the electronic and paper questionnaires on a 15-point Likert scale, ranging from −7 (a very great deal worse) to +7 (a very great deal better).^13^

### Study procedures

A study-specific online module was designed within the Zorgdraad integrated care IT system. The CAT and CCQ were designed to appear similar to the paper versions. The order in which the CAT or CCQ was presented to the patient was randomized to eliminate any completing fatigue effects. The time between loading of the webpage and submitting the form was recorded electronically. The easiness to complete, understand the questions, questionnaire preference, and importance of issues addressed were assessed using additional questions.

Within 1 week after completing the electronic version, the participant completed a paper copy of the CAT and the CCQ at their homes. In addition, the GRC was completed to assess stability of the disease. For comparison of the electronic and paper versions, only stable patients with a GRC score between −1 to +1 were included in the analysis.

### Sample size and statistical analysis

A sample size calculation using data from Ringbaek and colleagues^8^ with a power of 80% and a two-sided significance level of 5% indicated that 88 patients were needed to complete the study to show at least a 30 s difference in filling out time, which difference we considered clinically relevant.

Difference in time to complete the electronic version of the CAT and the electronic version of the CCQ was tested using a paired sample *t-*test. In the event assumptions of the *t*-test were violated, testing was conducted on logtransformed variables or, if unsuccessful, Mann–Withney U tests. Data were descriptively presented as medians with IQRs.

The differences between the electronic versions of the CAT and the CCQ regarding easiness to complete, importance of issues raised in questionnaires, and the importance of information for health-care provider were determined using paired sample *t*-tests, or, when assumptions were violated, paired sample *t*-tests on logtransformed variables. If logtransformation was unsuccessful Wilcoxon signed-rank tests were used. The intraclass correlation coefficient (ICC) and Bland–Altman plots were used to assess agreement between the electronic version and the paper version of the CAT and CCQ.

The study was registered at the Dutch Trial Register (NTR3384), and The University Medical Center Groningen Ethics Board approved the study.
